# Reversible mono‐ADP‐ribosylation of DNA breaks

**DOI:** 10.1111/febs.14297

**Published:** 2017-11-08

**Authors:** Deeksha Munnur, Ivan Ahel

**Affiliations:** ^1^ Sir William Dunn School of Pathology University of Oxford UK

**Keywords:** ADP‐ribose hydrolase, ADP‐ribosylation, DNA, DNA repair, PARP

## Abstract

Adenosine diphosphate (ADP)‐ribosylation is a chemical modification of macromolecules that plays an important role in regulation of quintessential biological processes such as DNA repair, transcription, chromatin remodelling, stress response, apoptosis, bacterial metabolism and many others. ADP‐ribosylation is carried out by ADP‐ribosyltransferase proteins, such as poly (ADP‐ribose) polymerases (PARPs) that transfer either monomer or polymers of ADP‐ribose onto the molecular targets by using nicotinamide adenine dinucleotide (NAD^+^) as a cofactor. Traditionally, proteins have been described as primary targets of ADP‐ribosylation; however, there has been growing evidence that DNA may be a common target as well. Here, we show using biochemical studies that PARP3, a DNA damage‐activated ADP‐ribosyltransferase, can mono‐ADP‐ribosylate double‐stranded DNA ends. ADP‐ribosylation of DNA mediated by PARP3 attaches a single mono‐ADP‐ribose moiety to the phosphate group at the terminal ends of DNA. We further show that mono ADP‐ribosylation at DNA ends can be efficiently reversed by several cellular hydrolases (PARG, MACROD2, TARG1 and ARH3). This suggests that mono ADP‐ribosylated DNA adducts can be efficiently removed in cells by several mechanisms.

**Abbreviations**
3′P: 3′ Phosphorylated terminal end
5′P: 5′ Phosphorylated terminal end
ADP: adenosine diphosphate
ADPr: ADP‐ribose
ALC1: amplified in liver cancer 1
APLF: aprataxin‐PNK‐like factor
ARH: ADP‐ribosylhydrolase
ART: ADP‐ribosyltransferases
CIP: alkaline phosphatase, calf intestinal
ds: double stranded
M: marker
MARylation: mono ADP‐ribosylation
noP: nonphosphorylated
NUDIX: nucleoside diphosphate linked to X‐moiety hydrolases
Oligo: oligomer
PARG: poly ADP‐ribose glycohydrolase
PARP: poly (ADP‐ribose) polymerase
PARP1 EQ: PARP1 E998Q mutant protein
PARylation: poly ADP‐ribosylation
TARG1: terminal ADP‐ribose protein glycohydrolase
β‐NAD^+^: β‐nicotinamide adenine dinucleotide

## Introduction

Adenosine diphosphate (ADP)‐ribosylation is a modification of macromolecules and is involved in the regulation of a number of processes such as DNA damage repair, transcription, chromatin structure, stress response, cell division and apoptosis 1, 2, 3, 4. Enzymes that can synthesise ADP‐ribose (ADPr) are called ADP‐ribosyltransferases (ARTs). All known ARTs utilise the β‐nicotinamide adenine dinucleotide (β‐NAD^+^) as a substrate to transfer ADPr on their molecular targets. The known ARTs can be broadly classified into several different families. The best understood are – (1) Diphtheria toxin‐like ADP‐ribosyltransferases (ARTDs), also commonly known as poly (ADP‐ribose) polymerases (PARPs), (2) Cholera toxin‐like ADP‐ribosyltransferases (ARTCs), bearing a related fold to PARPs 1, 2, and (3) Sirtuins, a family of NAD^+^‐dependent deacetylase with some members known to ADP‐ribosylate proteins 1, 2, 5, 6.

Poly (ADP‐Ribose) polymerases are one of the most widely studied ADP‐ribosyltransferase superfamily. There are 17 different mammalian PARPs localised in different cellular compartments such as nucleus, nucleolus, cytoplasm, plasma membrane, golgi, endoplasmic reticulum 7, 8, 9, 10. While mammalian PARPs have been most extensively studied for their role in DNA repair they have also been implicated in transcription, chromatin remodelling, unfolded‐protein response, host–virus interactions, cellular stress response and many more 1. Some members of the PARP family such as PARP1/2 and tankyrases can make poly (ADP‐ribose) (PAR) chains while most of the other members of the family can only attach a single unit of ADPr onto proteins and are referred to as mono‐ADP‐ribosyltransferases 11, 12, 13, 14.

Poly (ADP‐ribose) polymerase 1, PARP2 and PARP3 are all involved in maintaining genome stability and are swiftly recruited to the sites of DNA damage 15, 16, 17, 18. PARPs 1–3 are catalytically activated upon binding to DNA breaks, both single stranded and double stranded 14, 19, 20, 21, with PARP2 and PARP3 preferentially activated by DNA breaks with a 5′ phosphate 22. After binding DNA and subsequent activation, PARPs modify themselves with ADPr, a process known as automodification of PARPs. In addition, PARPs also catalyse ADP‐ribosylation of many effector proteins such as histones, chromatin remodellers, DNA repair factors such as Ku70/80, p53 14, 18, 23, 24, 25. DNA damage‐induced PARylation of histones causes decompaction of chromatin structure 1, 18, 26, 27. PARylation at the DNA damage site also acts as scaffolding platform for the recruitment of further DNA damage response effectors, such as XRCC1, APLF, ALC1 and Chk1 20, 26, 28, 29, 30, 31, 32, 33.

While both PARP1 and PARP2 are able to catalyse the attachment of PAR chains on proteins, PARP3 has a mono ADP‐ribosyltransferase activity 4, 12. PARP3 has a similar domain structure to PARP2, comprised of an N‐terminal extended domain, Trp‐Gly‐Arg (WGR) domain, helical domain (HD) and an ADP‐ribosyltransferase (ART) fold; but missing the additional three Zn fingers and BRCT domain present in PARP1 12, 14. PARP3 has been shown to play a role in double‐stranded DNA break repair. PARP3 partakes in the nonhomologous end joining (NHEJ) DNA repair pathway by accumulating APLF at damage sites, which in turn increases the retention of XRCC4/LigaseIV complex, and thereby facilitating DNA end ligation step 20. A proteomics‐based approach has shown PARP3 to associate with several proteins belonging to the NHEJ repair machinery such as DNA‐PKcs, PARP1, DNA Ligase IV and KU 70/80, along with DNA ligase III which is involved in base excision repair (BER) pathway 34. PARP3 has also been shown to interact with and ADP‐ribosylate Ku70 and Ku80, a function which helps to determine which double stranded break repair pathway should progress – NHEJ or homologous recombination (HR) 23. Recently, PARP3 has also been shown to promote single stranded DNA break repair, by accumulating at DNA damage sites induced by γ‐rays 24. Nicks in naked DNA were found to stimulate auto‐ribosylation of PARP3, however, nicks in mononucleosomes promoted trans‐ribosylation of histone H2B 24.

The process of ADP‐ribosylation has traditionally been considered a post‐translation modification of proteins. However, there has been emerging evidence that DNA ADP‐ribosylation maybe more common than previously thought. For example, ARTC family proteins pierisins and CARP‐1, found in cabbage butterfly and edible clams, respectively, exhibit ADP‐ribosylation activity on guanine nucleotide of DNA 35, 36, 37. Recently, bacterial toxin DarT was shown to ADP‐ribosylate single‐stranded DNA on thymidine bases in a sequence specific manner 38. Very recent experimental data on PARP1 and PARP2 have shown that these PARPs can also PARylate phosphorylated DNA termini *in vitro* 39.

Adenosine diphosphate‐ribosylation is usually a reversible modification and a number of enzymes have been described that can hydrolase it. The known mammalian ADPr hydrolase enzymes can be broadly classified as (1) macrodomain containing or (2) DraG‐like fold containing hydrolases. The best characterised macrodomain containing hydrolases are poly (ADP‐ribose) glycohydrolase (PARG), MacroD1, MacroD2 and terminal ADP‐ribose glycohydrolase 1 (TARG1). While these belong to same family, their specificities and reaction mechanisms are different 3. PARG, one of the most widely studied hydrolases, has been shown to rapidly catalyse the cleavage of PAR chains by hydrolysing the *O*‐glycosidic bond between the ADPr units, but is unable to hydrolyse the terminal ADP‐ribose unit linked directly to a protein 40, 41, 42, 43. TARG1, MacroD1 and MacroD2 hydrolyse mono‐ADPr at glutamic or aspartic acid residues 44, 45, 46, 47 and can also hydrolyse *O*‐acetyl‐ADP‐ribose *O*AADPr, a byproduct of sirtuins activity 48, 49 ADP‐ribosylhydrolase 1‐3 (ARH1‐3) are mammalian homologues containing the DraG‐like fold. ARH3 has been shown to hydrolyse PAR chains 50 and the *O*AADPr 51. Recently, it was shown that ARH3 is sufficient and necessary for efficient removal of ADP‐ribosylation on serine residues 52, a modification catalysed by PARP1/HPF1 and PARP2/HPF1 complexes 25, 53. ARH1 specifically removes ADPr from arginine residues 54. ARH2, on the other hand, has no known hydrolase activity. In addition to the above mentioned mammalian hydrolases, two completely unrelated protein families – (1) nucleoside diphosphate linked X‐moiety hydrolase (NUDIX) family proteins – hNUDT16 and *E. coli* RppH and (2) ectonucleotide pyrophosphate/phosphodiesterase (ENPP) family proteins – ENPP1 and snake venom phosphodiesterase (svPDE1) can also catalyse removal of MAR or PAR signal by hydrolysis of ADPr phosphodiester bonds 55, 56, 57, 58. The only known examples of enzymes performing reversal of DNA ADP‐ribosylation are DarG antitoxin partner of DarT DNA ADP‐ribosyltransferase 38 and PARG in removal of DNA PARylation catalysed by PARP1/2 39.

Research in the DNA ADP‐ribosylation field is still in its early stages. Until now only mammalian PARP1/2 were found to PARylate DNA ends. We wanted to examine whether some other mammalian PARPs could also modify DNA, especially the mono ADP‐ribosyltransferase representatives of this family. Here, we demonstrate, using biochemical assays, efficient mono–ADP‐ribosylation of DNA by PARP3. DNA ADP‐ribosylation by PARP3 is catalysed on the terminal phosphate moiety of DNA, with clear preference for 5′ ends of DNA double‐stranded breaks. We also show DNA ADP‐ribosylation catalysed by PARP3 can be reversed by several human hydrolases, of which PARG is most efficient. This study shows for the first time reversible mono ADP‐ribosylation of DNA by a mammalian PARP.

## Results

### Mono ADP‐ribosylation of DNA terminal ends by PARP3

**Figure 1:**
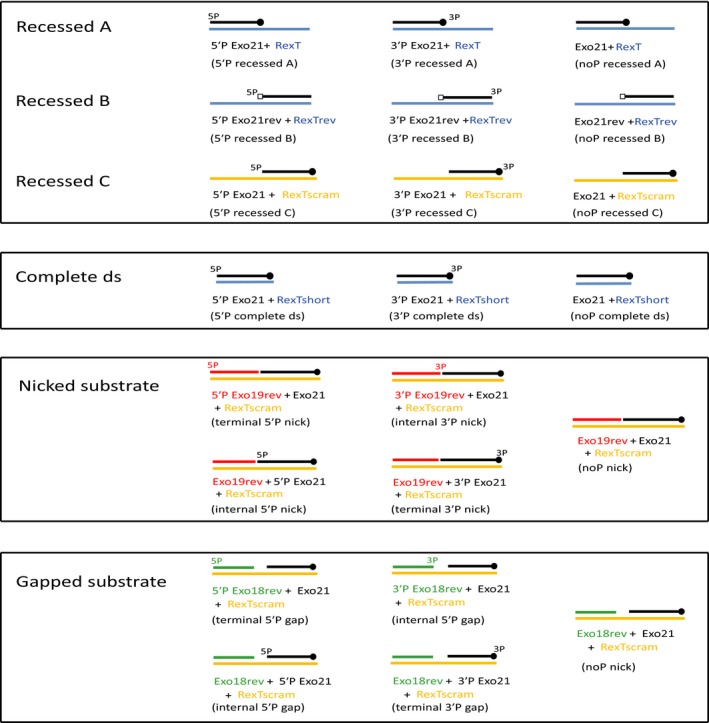
Schematic representation of DNA substrates used in this manuscript. Under each DNA substrate are the names of the oligonucleotides annealed together to make that particular substrate. Written within brackets underneath are the names they are referred to as in the study.

Recently, it was shown that PARP1 and PARP2 can modify terminal ends of DNA oligomers (oligos) by addition of poly ADP‐ribose chains, *in vitro* 39. We wanted to see if any other human PARPs could also ADP‐ribosylate DNA. One obvious candidate is PARP3 that has been implicated in DNA repair. In addition to PARP3, we also chose mono‐ARTs PARP10 59 and a point mutant of PARP1 that only catalyses mono ADP‐ribosylation – PARP1 E998Q 60, 61. To test DNA ADP‐ribosyltransferase activity of these PARPs we first used a DNA substrate that was previously shown to be a good substrate for PARP1‐ and PARP2‐mediated PARylation of DNA 39. This substrate is made of a phosphorylated short 21mer oligo annealed with a nonphosphorylated long 40mer oligo to create a recessed double stranded (ds) substrate with 19 nucleotide overhang at 3′ end of the short oligo. In the experiments henceforth we refer to this substrate as the recessed A substrate. In addition to the recessed A substrate we also designed several other substrates – recessed B, recessed C, complete ds, nicked and gapped substrates. The different substrates designed for this study are shown in Fig. 1 along with the oligos used to make them. The oligo sequences are listed in Table 1.

**Table 1:** Sequence of oligonucleotides used in this study

Name	Sequence (5′→3′)
5′P Exo21	[Phos] GTGGCGCGGAGACTTAGAGAA
3′P Exo21	GTGGCGCGGAGACTTAGAGAA [Phos]
Exo21	GTGGCGCGGAGACTTAGAGAA
5′P Exo21rev	[Phos] AAGAGATTCAGAGGCGCGGTG
3′P Exo21rev	AAGAGATTCAGAGGCGCGGTG [Phos]
Exo21rev	AAGAGATTCAGAGGCGCGGTG
5′P Exo18rev	[Phos] CCTTAAGGGGCGCGGTTT
3′P Exo18rev	CCTTAAGGGGCGCGGTTT [Phos]
Exo18rev	CCTTAAGGGGCGCGGTTT
5′P Exo19rev	[Phos] CCTTAAGGGGCGCGGTTTA
3′P Exo19rev	CCTTAAGGGGCGCGGTTTA [Phos]
Exo19rev	CCTTAAGGGGCGCGGTTTA
5′P Exo21_DarT	[Phos] GTGGCGCGGAGACTTTCAGAA
Exo21_DarT	GTGGCGCGGAGACTTTCAGAA
RexT	GGAATTCCCCGCGCCAAATTTCTCTAAGTCTCCGCGCCAC
RexTrev	CACCGCGCCTCTGAATCTCTTTAAACCGCGCCCCTTAAGG
RexTscram	TTCTCTAAGTCTCCGCGCCACTAAACCGCGCCCCTTAAGG
RexTshort	TTCTCTAAGTCTCCGCGCCAC

**Figure 2:**
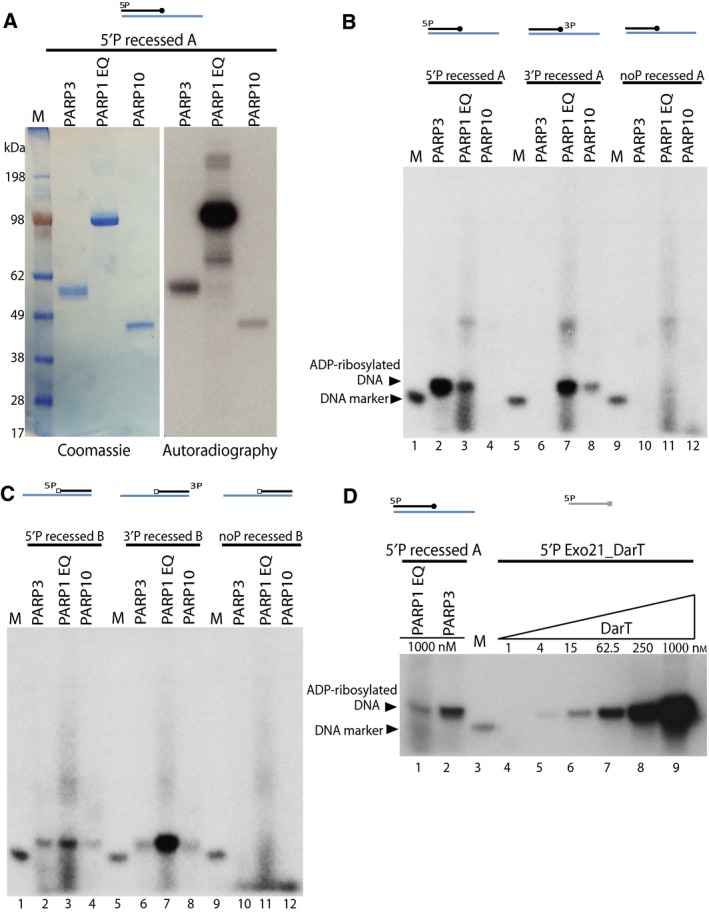
Mono ADP‐ribosylation of DNA ends by PARPs. (A) Auto‐ADP‐ribosylation of PARP3, PARP1 E998Q and PARP10 – left panel shows the coomassie staining and right panel shows the autoradiography of the SDS/PAGE gel. (B) Mono‐ADP‐ribosylation of phosphorylated or nonphosphorylated recessed A DNA substrate catalysed by PARP3, PARP1 E998Q or PARP10 as seen from autoradiograph of a denaturing urea PAGE gel (C) ADP‐ribosylation of phosphorylated or nonphosphorylated recessed B DNA substrate by PARP3, PARP1 E998Q or PARP10. (D) Comparison of DNA ADP‐ribosylation activity of PARP3 and PARP1 E998Q on 5′P recessed A substrate against DNA ADP‐ribosylation of DarT on single stranded Exo21_DarT oligo.

Before we tested the PARPs for DNA ADP‐ribosylation activity, we performed the automodification assays to confirm their activity. Automodification assays showed all three PARPs used for this study were active (Fig. 2A).

Poly (ADP‐ribose) polymerase 1 can PARylate DNA ends 39, so given the expected mono‐ADP‐ribosylation activity of PARP3 and PARP10 we used PARP1 E998Q as a positive control for mono ADP‐ribosylation of DNA 39, 60, 61. First set of DNA substrates – recessed A substrate was prepared with or without phosphate at terminal end of shorter 21mer oligo for DNA ADP‐ribosylation assay (Fig. 1). The recessed A DNA substrates were tested with mono ADP‐ribosyltransferase PARPs – PARP3, PARP1 E998Q and PARP10, using ^32^P labelled NAD as an ADP‐ribose donor. Size marker (phosphorylated 21mer) was used for all *in vitro* assays. Autoradiography of the denaturing urea PAGE gel revealed PARP3 could efficiently modify 5′P recessed A DNA substrate (Fig. 2B, lane 2) but was unable to modify the 3′P recessed A substrate (Fig. 2B, lane 6). PARP1 E998Q mutant showed a preference for 3′P recessed A substrate over 5′P recessed A substrate (Fig. 2B, lane 3 and 7). PARP10 showed very weak ADP ribosylation with 3′P recessed A substrate but did not modify the 5′P recessed A oligo (Fig. 2B lane 4 and 8). When nonphosphorylated recessed A substrate was tested with PARP3, PARP E998Q and PARP10 no DNA ADP‐ribosylation was observed (Fig. 2B lane 10‐12). This strongly indicates that the ADP‐ribosylation of DNA is taking place on the DNA terminal phosphate group.

We further tested the mono‐ARTs with recessed B substrate (Fig. 1), where the 3′ terminal end of short 21mer oligo formed the blunt end and the 19 nucleotide overhang was at the 5′ end of DNA. PARP1 E998Q protein modified both 5′P and 3′P recessed B substrate with preference for the phosphate group at the 3′ end (Fig. 2C, lane 3 and 7). PARP3 and PARP10 both showed weak DNA modification at both 5′P and 3′P ends (Fig. 2C, lane 2, 4, 6 and 8) and nonphosphorylated recessed B substrate was not modified by any PARPs tested (Fig. 2C, lane 10–12). PARP3 was able to weakly modify 3′ end of DNA when the phosphate group was at the blunt end DNA (Fig. 2C, lane 6). However, changing the 5′ terminal phosphate from the blunt end (as seen with 5′P recessed A) to 5′ overhang end (as seen with 5′P recessed B substrate) caused a substantial loss in DNA ADP‐ribosylation signal (lane 2 of Fig. 2B,C). This result suggests that PARP3 preferentially mono ADP‐ribosylates DNA on 5′P at the blunt end of DNA, however, it can also ADP‐ribosylate the phospho group on the 5′ ends with an overhang. PARP3 can weakly ADP‐ribosylate 3′P but only when the phosphate group is present at the blunt end of DNA. PARP1 E998Q, on the other hand preferentially ADP‐ribosylates 3′P over 5′P end without preference for blunt or overhang ends.

Next, we wanted to compare the MARylation of DNA by PARP3 to known DNA mono‐ADP‐ribosyltransferase protein. For this purpose we used the bacterial toxin DarT, which has been shown to ADP‐ribosylate thymidine base in a sequence‐specific manner on a single‐stranded DNA 38. We designed a 21mer oligo nucleotide similar to the ones’ used in recessed A substrate and changed two nucleotides to incorporate a single DarT modification motif – TNTC, referred henceforth as 5′P Exo21_DarT oligo. DarT has been shown to strongly mono‐ADP‐ribosylate DNA, hence we did a titration of DarT to compare the DNA modification efficiencies by DarT to PARP3 and PARP1 E998Q protein. PARP3 was more than one order of magnitude less efficient than DarT at DNA ADP‐ribosylation in these conditions (Fig. 2D lane 2 versus lane 7). In addition, this result also suggests that PARP3 is only adding a single ADPr to DNA because the product of PARP3 and DarT ADP‐ribosylation has the same mobility in the gel. PARP1 E998Q protein weakly modifies 5′P recessed A substrate (Fig. 2B, lane 3 and Fig. 2D, lane 1) and has much lower DNA ADP‐ribosylation activity when compared against DarT (Fig. 2D, lane 1 and lane 6).

### Characterisation of DNA ADP‐ribosylation

**Figure 3:**
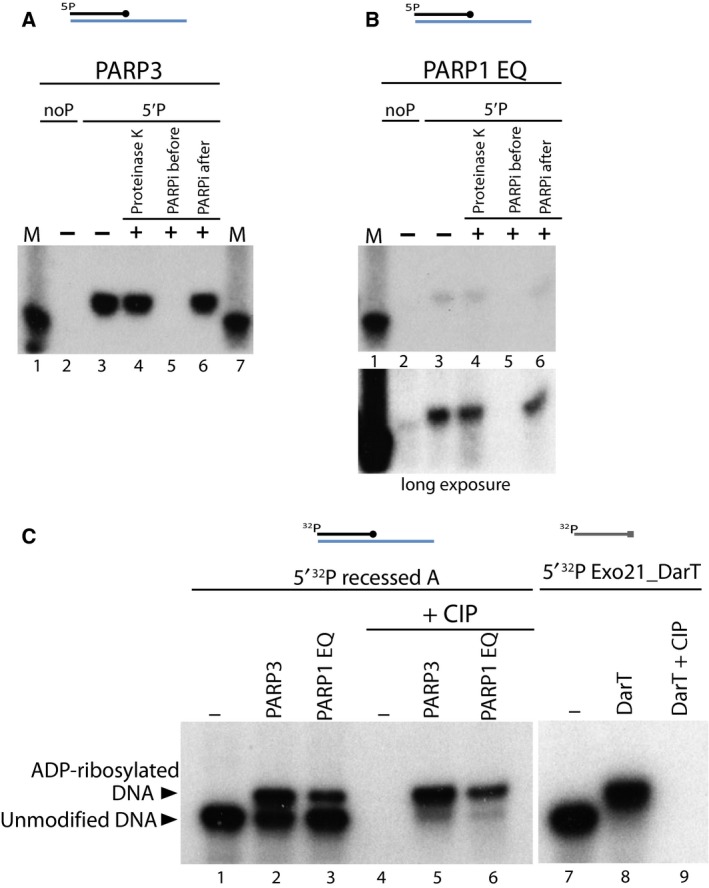
Characterisation of DNA ADP‐ribosylation activity. (A) ADP‐ribosylation of 5′ phosphorylated recessed A substrate catalysed by PARP3 is protected against Proteinase K and SDS treatment. Some reactions were treated with Olaparib, a PARP inhibitor added before the start of reaction or after the ADP‐ribosylation reaction was complete. (B) ADP‐ribosylated 5′P recessed A substrate catalysed by PARP1 E998Q is resistant to Proteinase K and PARP inhibitor treatment. The lower panel indicates a longer exposure of autoradiography. (C) PARP3 and PARP1 E998Q catalysed DNA ADP‐ribosylation is protected against phosphatase activity of CIP. ADP‐ribosylation of 5′ ^32^P recessed A substrate by PARP3 and PARP1 E998Q protein further treated with or without CIP on left panel. Right panel indicates ADP‐ribosylation of 5′ ^32^P radiolabelled Exo21_DarT oligo by toxin DarT protein further treated with CIP.

We designed further experiments to prove that the product of ADP‐ribosylation reaction is indeed ADP‐ribosylated DNA and that this depends on the catalytic activity of PARP enzymes. We used the 5′P or noP recessed A substrate for this experiment. ADP‐ribosylation by PARP3 and PARP1 E998Q was only observed in the presence of phosphorylated DNA (Fig. 3A,B, lane 3) and not with non‐phosphorylated DNA substrate (Fig. 3A,B, lane 2). The ADP‐ribosylation signal obtained with both PARP3 and PARP1 E998Q reaction was resistant to Proteinase K and SDS treatment (Fig. 3A,B, lane 4). This indicates the signal observed is not result of protein contamination or protein ADP‐ribosylation but is indeed modification of DNA mediated through transfer of single ADP ribose unit from ^32^P labelled NAD by PARP3 and/or PARP1 E998Q. Olaparib, an inhibitor of PARP enzyme was added before the start of the reaction, which caused total loss of DNA ADP‐ribosylation signal by both PARP3 and PARP E998Q protein (Fig. 3A,B, lane 5) confirming that the catalytic activity of PARPs is required for DNA ADP‐ribosylation. Olaparib did not affect DNA modification signal when it was added after the ADP‐ribosylation reaction took place (Fig. 3A,B, lane 3 versus lane 6).

In light of the finding above of 5′P DNA ADP‐ribosylation mediated by PARP3 or PARP1 E998Q, we wanted to confirm the attachment site of ADP‐ribose unit. For this study we used the 5′ ^32^P radiolabelled recessed A oligo with unlabelled β‐NAD^+^ and PARPs. Using this radiolabelled oligo we could see that around 50% of DNA was ADP‐ribosylated by PARP3 under our conditions (Fig. 3C lane 1 versus lane 2). In comparison, PARP1 E998Q modified a smaller fraction of DNA (Fig. 3C, lane 3). DarT on the other hand was able to completely modify ^32^P labelled Exo21_DarT substrate (Fig. 3C, lane 7 and 8). At the next step, an aliquot of the reactions was further treated with alkaline calf intestinal phosphatase (CIP), which dephosphorylates the 5′ and 3′ ends of DNA. In the absence of any PARP protein, CIP was able to catalyse complete dephosphorylation of ^32^P‐labelled phospho group at the 5′ terminal end of DNA, indicated by loss of signal in Fig. 3C, lane 4. In the presence of PARPs, when the DNA has been ADP‐ribosylated, CIP was unable to dephosphorylate the upward shifted ADP‐ribosylated DNA band while the signal corresponding to the lower unmodified DNA was removed (Fig. 3C, lane 5 and 6). These data further confirm that the ADP‐ribosylation of DNA by PARPs occurs on the phospho group at the terminal ends of DNA which protects the phosphate moiety from dephosphorylation activity of CIP. In case of DarT‐mediated DNA ADP‐ribosylation where the modification is on the thymidine base (thus leaving the terminal ^32^P labelled end accessible to CIP treatment) the DNA radioactive signal is completely lost upon phosphatase treatment (Fig. 3C, lane 9).

### ADP‐ribosylation of DNA by PARP3 preferentially targets dsDNA ends

We wanted to further analyse the specificity of PARP3 towards different DNA substrates and tested recessed A/B/C, nicked, gapped and complete ds DNA structure with phosphates at different terminal ends (Fig. 1). From earlier experiments it was clear that PARP3 could only ADP‐ribosylate recessed A DNA on the phosphate group at the 5′ end and not 3′ end (Fig. 2B, lane 2 and 6). We wanted to test if this was because PARP3 could only ADP‐ribosylated DNA at 5′ end or because PARP3 was unable to access the phosphate group at 3′end due to the 19 nucleotide overhang at the 3′ end as seen in recessed A substrate. To resolve this question, we designed 2 additional recessed DNA substrates, where the overhang of the longer 40mer annealing strand was at the 5′end of the shorter 21mer oligo. We called these two sets of recessed substrates as – 1) recessed B and 2) recessed C substrate. The difference between these two substrates being recessed B substrate uses completely different short 21mer oligo sequence while the recessed C substrate uses the same short 21mer oligo sequence as recessed A substrate. In each of the recessed A/B/C substrate only the short 21mer oligo contains 5′P, 3′P or noP group attached (as seen in Fig. 1).

**Figure 4:**
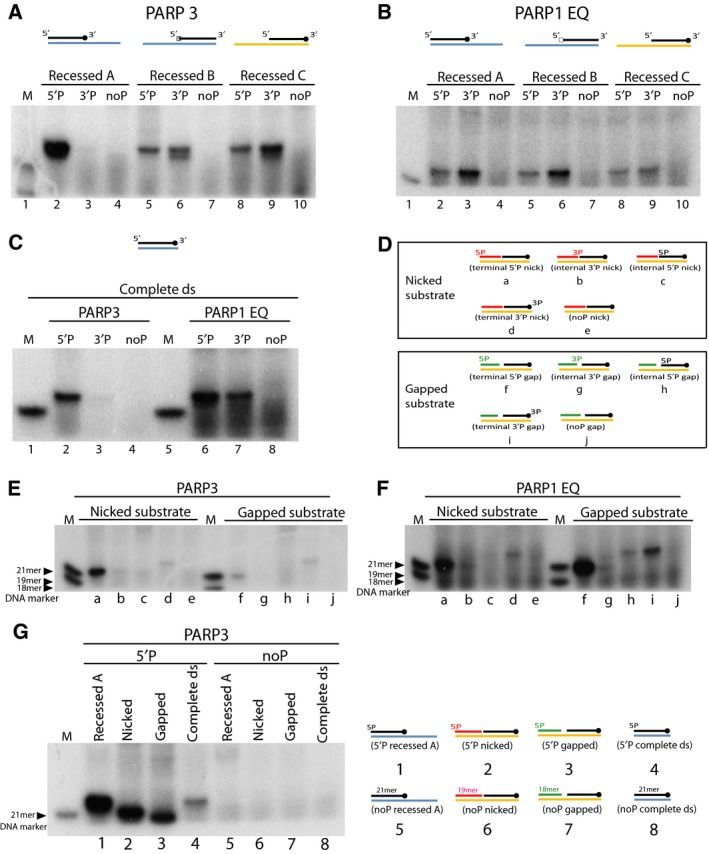
PARP3 and PARP1 E998Q preferentially ADP‐ribosylate double stranded DNA ends. (A) ADP‐ribosylation of 5′ or 3′ phosphorylated or nonphosphorylated recessed A/B/C substrate by PARP3 (B) ADP‐ribosylation of different recessed A/B/C substrates catalysed by PARP1 E998Q protein. (C) ADP‐ribosylation of 5′P or 3′P or noP complete ds DNA catalysed by PARP3 and PARP1 E998Q. (D) Schematic representation of nicked and gapped substrates with phosphates attached at different ends of the oligo. The different substrates are annotated from a to j and also referred as lane marking in panels E and F below. (E) ADP‐ribosylation by PARP3 on nicked and gapped DNA substrates. (F) PARP1 E998Q mediated DNA ADP‐ribosylation of nicked and gapped substrate. Lanes in panel E and F are annotated according to the substrate used as shown in panel D. (G) Comparison of the relative ADP‐ribosylation efficiencies of different 5′ phosphorylated DNA substrates – recessed A, nicked, gapped and complete ds. Lanes in the left panel are annotated (1–8) according to the substrates used as shown in right panel.

Both PARP3 and PARP1 E998Q were tested with the different recessed A/B/C substrates. PARP3 was able to ADP‐ribosylate 5′P recessed A substrate strongly but not the 3′P recessed A substrate (Fig. 4A, lane 2 and 3). However, PARP3 was able to modify phosphorylated DNA at the 3′ blunt end of the substrate as seen in recessed B and recessed C substrate (Fig. 4A, lane 6 and 9), but not when the 3′P was at the 3′ overhang end as seen with recessed A substrate (Fig. 4A, lane 3). While PARP3 was able to modify the phospho group at the 5′ overhang end, as seen with recessed B and recessed C substrate (Fig. 4A, lane 5 and 8), the modification was much weaker when compared to ADP‐ribosylation seen with 5′P recessed A substrate where the phosphate was at the blunt end of the substrate (Fig. 4A, lane 2). These data strongly suggest that while phosphate moiety at 5′ end can modified at both blunt and overhang end; the 5′P blunt end was the preferred substrate for PARP3. On the other hand 3′P end could only be modified when the 3′ end formed a blunt end. This would indicate strong preference for blunt ended substrate for DNA ADP‐ribosylation by PARP3. In contrast, PARP1 E998Q could ADP‐ribosylate DNA at both 5′P and 3′P, with a marginal preference towards 3′P irrespective of phospho moiety being at the blunt or overhang end of the different recessed substrates (Fig. 4B).

Next, the simple (complete) double‐stranded (ds) substrate with or without phosphate group at different ends were tested with PARP3 and PARP1 E998Q. PARP3 ADP‐ribosylated complete ds DNA at 5′P end and weakly modified 3′P end (Fig. 4C, lane 2 and 3). PARP1 E998Q modified both phosphorylated ends of the complete ds substrate (Fig. 4C, lane 6 and 7). The results from both recessed A/B/C and complete ds substrate would indicate that phosphorylation at the 5′ blunt end is a preferred acceptor site for PARP3 to attach the ADP‐ribose unit.

We also tested nicked and gapped substrate (Fig. 1 and Fig. 4D). The substrates were designed such that there was only a single phosphate group at either terminal or internal ends of the nick or gap substrate (Fig. 4D). PARP3 was able to ADP‐ribosylate both nicked and gapped substrate. ADP‐ribosylation was only catalysed at terminal 5′P or terminal 3′P end of both nicked and gapped substrates (Fig. 4E, lane a, d, f and i) and not the 5′ or 3′ phosphate group present internally at the nick and gap (Fig. 4E, lane b, c, g and h). Terminal 5′P nicked substrate (Fig. 4E, lane a) was preferred over terminal 5′P gapped and both terminal 3′P substrates (Fig. 4E, lane d, f and i). In contrast, PARP1 E998Q showed preference towards gapped over nicked substrate (Fig. 4F, lane a and f). PARP1 E998Q also preferentially ADP‐ribosylated the terminal 5′P over terminal 3′P ends of nick and gap substrate (Fig. 4F, lane a, d, f and i). PARP1 E998Q was able to very weakly ADP‐ribosylate internal 5′P gapped substrate (Fig. 4F, lane h). There has been evidence showing PARP3 is preferentially activated by 5′P nicked DNA 22, it is therefore interesting to note that while using our nicked substrate, we did not see any ADP‐ribosylation of DNA at the internal phosphorylation site (Fig. 4E, lane b and c), a modification which we postulate could be prevented due to steric hindrance or because the internal phospho sites are involved in automodification of PARP3 and thus are unable to undergo ADP‐ribosylation themselves.

For each of the DNA substrates (recessed, complete ds, nicked and gapped) tested above, terminal 5′ end phosphate was preferred site for attachment of mono‐ADP‐ribose unit by PARP3. Comparing the relative modification efficiencies of these substrates showed that the complete ds substrate was in comparison less efficiently modified (Fig. 4G, lane 1‐4).

### Mono‐ADP‐ribosylation of DNA is a reversible process

Having characterised the ADP‐ribosylation of DNA by mono ADP‐ribosyltransferease PARPs, we wanted to test whether this DNA modification is a reversible process. For this we tested most of the known mammalian ADPr hydrolases: PARG, TARG1, MacroD2 and ARHs 1–3. We also tested human NUDT16 that was known to cleave ADP‐ribosylation into phospho‐ribosylation 55.

**Figure 5:**
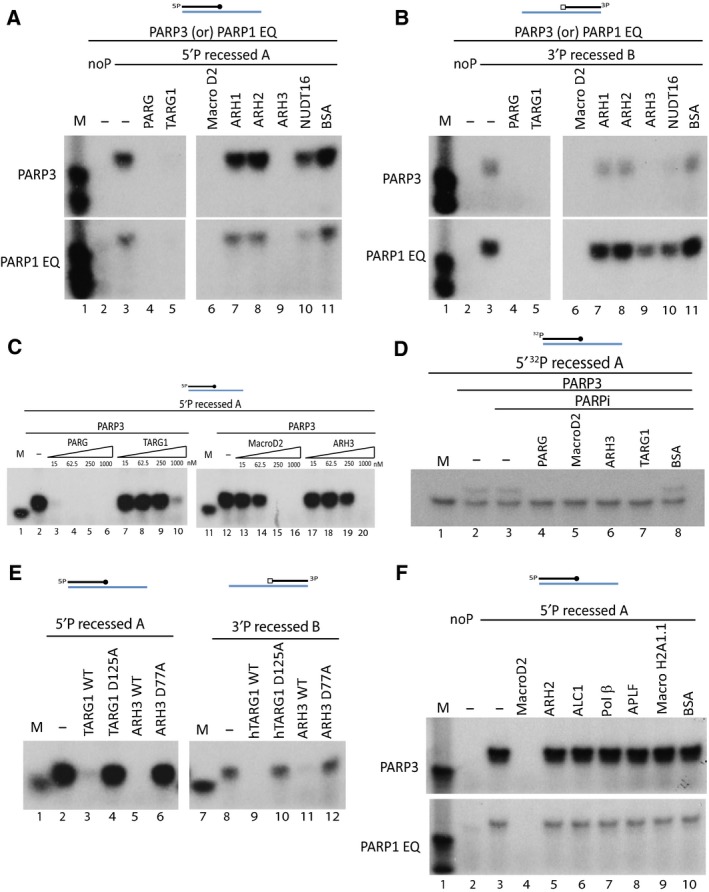
Mono ADP‐ribosylation of DNA is a reversible process. (A) Reversal of ADP‐ribosylated 5′P recessed A substrate by several different hydrolases. (B) ADP‐ribosylated 3′P recessed B substrate is treated with different hydrolases. For both (A) and (B) ADP‐ribosylation of DNA is catalysed by PARP3 (in top panel) or PARP1 E998Q (in bottom panel). (C) PARP3 catalysed ADP‐ribosylation of DNA is subjected to different concentration of hydrolase active in removing DNA ADP‐ribosylation – PARG, TARG1, MacroD2 and ARH3. Hydrolase concentration is titrated from 15 nm to 1000 nm. (D) Mono‐ADP‐ribosylation of ^32^P labelled DNA substrate is reversed by various hydrolases. (E) Removal of PARP3 mediated ADP‐ribosylation on 5′P recessed A (left panel) and 3′P recessed B (right panel) by wild‐type or point mutant of TARG1 and ARH3. (F) Lack of hydrolytic activity of ADP‐ribosylation‐related proteins – ARH2, ALC1, Polymerase β, APLF and macroH2A1.1 on ADP‐ribosylated DNA substrate catalysed by PARP3 (top panel) and PARP1 E998Q (bottom panel).

The preferred ADP‐ribosylation DNA substrate of PARP3 (5′P recessed A) was used to test different hydrolases. As a control step we also included the noP recessed A substrate that we know from earlier experiments (Figs 2, 3, 4) could not be ADP‐ribosylated by PARP activity (Fig. 5A, lane 2). The 5′P recessed A DNA was ADP‐ribosylated by PARP3 or PARP1 E998Q (Fig. 5A, lane 3) and further used as a substrate for hydrolase enzymes. PARG, TARG1, MacroD2 and ARH3 catalysed efficient removal of DNA ADP‐ribosylation (Fig. 5A, lane 4–6 and 9). On the other hand NUDT16 protein only very weakly cleaved DNA ADP‐ribosylation (Fig. 5A, lane 10), while ARH1, ARH2 and BSA control were unable to hydrolyse the ADPr DNA adduct (Fig. 5A, lane 7, 8 and 11). We also tested the hydrolase enzymes against the ADP‐ribosylated 3′P substrate, to assess if any hydrolase enzymes showed substrate specificity in reversing ADP‐ribosylation at 5′ or 3′ phosphorylated DNA ends. Similar to 5′P recessed A substrate; PARG, TARG1 and MacroD2 were able to reverse ADP‐ribosylation at 3′P end (Fig. 5B, lane 4–6). NUDT16 showed poor activity against DNA ADP‐ribosylation at 3′P (Fig. 5B, lane 10).

Next, we wanted to compare the relative efficiency of the hydrolases tested in Fig. 5A and 5B at removing DNA ADP‐ribosylation. To discern this, we did a titration of the amount of different hydrolases to compare their hydrolytic efficiency on ADP‐ribosylated 5′P recessed A substrate (made by PARP3). Our results indicated that PARG was the most active hydrolase in these assays followed by MacroD2, ARH3 and TARG1 sequentially (Fig. 5C). To further confirm that the removal of ADP‐ribosylation signal was due to de‐ADP‐ribosylation activity of hydrolases, we mono‐ADP‐ribosylated ^32^P labelled oligo in the presence of PARP3 (Fig. 5D, lane 2 and 3) and used it as substrate for hydrolase enzymes. Our results clearly show complete reversal of ADP‐ribosylated oligo in the presence of PARG, MacroD2, ARH3 and TARG1 converting it into the faster‐migrating unmodified DNA signal (Fig. 5D, lane 4‐7). This result also excludes the possibility of nonspecific nuclease contamination in reaction.

We also tested the hydrolases which had relatively weaker activities on removing DNA ADP‐ribosylation – TARG1 and ARH3 to confirm that their reaction is indeed enzymatic. We used the characterised inactive mutants of TARG1 D125A 44 and ARH3 D77A 52. Point mutants of both TARG1 and ARH3 were unable to remove ADP‐ribose units transferred onto phosphorylated DNA by PARP3 (Fig. 5E, lane 4, 6, 10 and 12). This result also suggested that the hydrolases use the same catalytic residues for DNA‐ and protein‐de‐ADP‐ribosylation reactions.

We also tested proteins that contain domains implicating their involvement in ADP‐ribosylation activity, such as macrodomain‐containing ALC1, histone macroH2A1.1, PBZ domain containing APLF, DraG family protein ARH2 and DNA polymerase β, which is known to release 5′ terminal deoxyribose phosphate residue from AP sites. None of the above mention proteins were able to remove ADP‐ribose moiety transferred to 5′P recessed A DNA substrate by PARP3 or PARP1 E998Q (Fig. 5F, lane 5–10).

## Discussion

**Figure 6:**
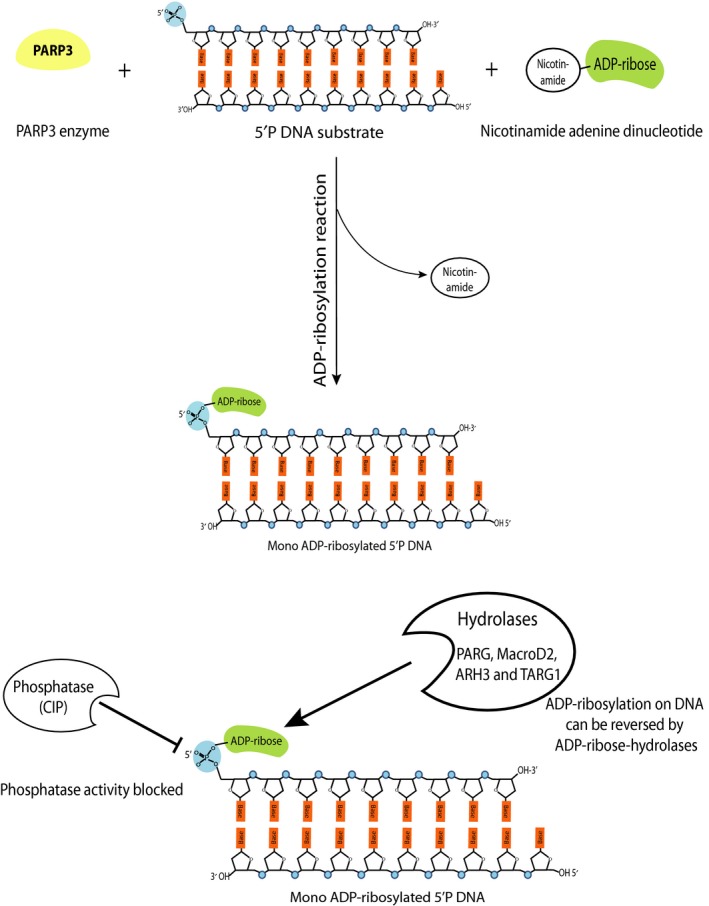
Schematic illustration depicting mono‐ADP‐ribosylation of 5′ phosphorylated double‐stranded DNA end by PARP3 and the reversal of ADP‐ribosylation by the ADP‐ribose hydrolases such as PARG, MacroD2, ARH3 and TARG1. ADP‐ribosylated DNA ends are protected against the activity of phosphatases such as CIP.

We present data that prove for the first time, human PARPs can efficiently mono ADP‐ribosylate DNA *in vitro*. PARP3, in particular, can transfer a single unit of ADP‐ribose onto phosphorylated ends of DNA (Figs 3A and 6). The preferred DNA substrate for mono ADP‐ribosylation is the phosphate group on the 5′ blunt end of DNA (Figs 4 and 6). This fits with recent data demonstrating that PARP3 catalytic activity is primarily activated by 5′P DNA substrates 22. The preference of PARP3 for the blunt end of dsDNA could suggest that DNA ADP‐ribosylation signalling might have a function in double‐stranded DNA repair, a hypothesis that we find plausible given PARP3′s published activities with this type of DNA damage response 20, 23, 34.

Based on our *in vitro* results, ADP‐ribosylation of DNA ends by PARP3 is a reversible process that could be undertaken by several known human ADP‐hydrolases (Fig. 5; schematically presented in Fig. 6). We found that PARG is the most efficient hydrolase at catalysing the reversal of ADP‐ribosylation on DNA with other hydrolases such as MacroD2, ARH3 and TARG1 also reversing DNA ADP‐ribosylation.

While the results presented herein are based on *in vitro* assays and do not provide any evidence for a physiological function for PARP3‐mediated DNA ADP‐ribosylation, we nevertheless offer some hypotheses for the possible mechanistic role for this DNA‐based modification. ADP‐ribosylation of DNA ends could serve as a mechanism to protect DNA ends from uncontrolled degradation by nucleases, indeed we did observe that ADP‐ribosylated 5′P DNA was protected from phosphatase activity of CIP (Fig. 3C and Fig. 6). Alternatively, ADP‐ribosylation at the DNA end could serve as a signal for specific processes such as apoptosis, by blocking DNA ends and thereby limiting access to DNA repair factors to damage sites.

However, it is equally possible that that this ADP‐ribosylation activity of DNA by PARP3 is an erroneous activity of the PARPs, thereby creating DNA lesions (adducts) similar to DNA adenylates that are formed during abortive DNA ligation events 62. In both cases, 5′ phosphorylated DNA traps a nuclear enzyme to produce nucleotide DNA adducts. In the case of DNA 5′P adenylates, these are processed by Aprataxin, a DNA repair factor responsible for direct reversal of these lesions restoring the conventional 5′P DNA end which can then be ligated in the presence of the DNA ligase 62, 63. Similarly, ADP‐ribose‐hydrolases could serve as repair enzymes by reversing ADP‐ribosylation from the phosphorylated DNA ends.

Altogether our results, along with other studies of DNA ADP‐ribosylation 35, 36, 38, 39, strongly suggest that DNA may represent a common target for ADP‐ribosylation in cells. In addition, we find that PARP3 mediated DNA mono‐ADP‐ribosylation at DNA breaks is reversible, and suggest that this may be important for efficient DNA repair.

## Materials and methods

### Plasmid and protein purification

Genes encoding for PARP3 and PARP1 E998Q were expressed in pDEST17 and pET28 vector, respectively, and were purified as previously described 22, 44. In short, the bacterial pellets were lysed using homogeniser and subjected to three‐step purification process – first nickel column to separate his‐tagged protein from bacterial lysate followed by heparin column to remove contaminant DNA and finally size exclusion chromatography column.

All the protein listed below were gifts from other members of lab. *Taq*DarT was expressed in pBAD vector, transformed into BL21 strains, induced with arabinose and purified using TALON affinity resin (Clontech) as described earlier 38. GST‐tagged PARP10 cd (aa818‐1025) was cloned in pGEX‐4T1 and purified as described 57. MacroD2 45, macrodomain of macroH2A1.1 45, TARG1 44, PARG 64, NUDT16 55, APLF 30 and ARH 1‐3 52 were purified as described earlier. Macrodomain of ALC1 was gifted from Dragana Ahel lab. Human polymerase β protein was purchased from Trevigen.

### Oligonucleotides

Oligonucleotides used in this study were adapted from an earlier described study 39 and are listed in Table 1. Oligonucleotides were commercially purchased with or without phosphate label at 5′ or 3′ ends from Life Technologies. Recessed A/B/C substrates were prepared with two sets of oligos – short 21mer oligo and annealing partner long 40mer oligo. The short 21mer oligo was designed with 5′ or 3′ or no phospho group linked. In the recessed A substrate, 5′ end of the short 21mer oligo formed the blunt end, while recessed B and C formed the blunt end at the 3′ end of short 21mer oligo. The difference between recessed B and C substrate was the sequence of short 21mer oligo, recessed C substrate had 100% sequence similarity to short 21mer oligo used in recessed A substrate. The annealing partner and oligo names used to create any given DNA substrate are shown in Fig. 1 and sequences for the oligos are listed in Table 1.

Complete ds substrate was designed similar to recessed A substrate, with same short 21mer oligo but the long 40mer annealing oligo was changed to complementary 21mer oligo (Fig. 1 and Table 1). We also prepared nicked substrates using short 21mer, shorter 19mer and long annealing 40mer oligo with phosphates are different ends of short 21mer or shorter 19mer DNA (Fig. 1 and sequences listed in Table 1). Gapped substrates were made similar to nicked substrate using short 21mer, shorter 18mer and long annealing 40mer oligo.

Oligonucleotides were diluted in 20 mm HEPES‐KOH (pH 7.6) and 50 mm KCl buffer. Complementary strands of DNA (as shown in Fig. 1) were annealed at 95 °C for 5 mins and then allowed to gradually cool down to room temperature.

50pmol Exo21 (short 21mer) oligo was radioactively labelled at 5′ end using T4 polynucleotide kinase (NEB) in the presence of γ^32^P ATP (Perking Elmer) and heated at 37 °C for 30 mins followed by heat inactivation at 65 °C for 20 mins. Radiolabelled oligo was further desalted on G25 column to remove any unincorporated ATP. Radiolabelled Exo21 was then annealed with complementary strand RexT (long 40mer) as described earlier. This radiolabelled oligo (5′ ^32^P recessed A substrate) was used as size marker and for the CIP experiments as indicated in figures. ^32^P‐labelled short 21mer and ^32^P‐labelled shorter 19mer or shorter 18mer were annealed with long 40mer and used as size marker when testing the nicked or gapped substrate respectively (Fig. 4E,F).

### Poly (ADP‐ribosyl)transferase auto‐modification assay

Automodification assay of PARPs were performed as described earlier 42. We used the 5′P recessed A DNA for the automodification assay (Fig. 1). Reaction mixture contained 1 μm protein (PARP3, PARP1 E998Q and PARP10), 2 μm DNA, 50 μm NAD (Trevigen), 10 kBq ^32^P‐labelled NAD (PerkinElmir), 50 mm Tris pH 7.5, 50 mm NaCl and 1 mm MgCl_2_ incubated at room temperature for 20 mins. Reactions were stopped by addition of 1 μm PARP inhibitor, Olaparib. The reactions were run on SDS/PAGE gel and analysed by autoradiography.

### DNA ADP‐ribosylation assay

DNA ADP‐ribosylation assays were performed as earlier described 39. In short, 10‐μL reaction mix was prepared in buffer containing 20 mm HEPES‐KOH (pH 7.6), 50 mm KCl, 5 mm MgCl_2_, 1 mm DTT and 100 μg·mL BSA. DNA substrate (10 μm) was added along with 1 μm protein, 50 μm NAD (Trevigen) and 50 kBq ^32^P labelled NAD (PerkinElmer) per reaction. When using ^32^P‐labelled DNA substrate, the reaction was activated with 1 mm NAD (Trevigen). Reactions were incubated at 37 °C for 30 mins and stopped by addition of 1 μm PARP inhibitor (olaparib) or by incubating the reactions with 50 ng·μL Proteinase K and 0.15% SDS at 50 °C for 30 mins. Samples were heated at 95 °C for 3 mins with 2X TBE Urea sample buffer (8 m urea, 20 μm EDTA pH 8.0, 2 μm Tris pH7.5 and bromophenol blue). The samples were loaded on a prerun denaturing urea PAGE gel composed of 20% (w/v) polyacrylamide, 8 m urea and 1X TBE. The gel was run at 10‐12W in 0.5X TBE buffer. The gel was dried under vacuum and visualised by autoradiography.

### DNA ADP‐ribose hydrolase assay

Hydrolysis of ADP‐ribosylated DNA was performed on reaction stopped with PARP inhibitor to abolish the activity of PARPs. 1 μm hydrolases enzymes were added per reaction unless otherwise stated. Reaction containing PARG or TARG1 (WT and D125A) were incubated at room temperature for 30 mins. While, reaction containing macroD2, ARH1, ARH2, ARH3 (wild‐type and D77N mutant), NUDT16, BSA, macrodomain of ALC1, Polymerase β, APLF or macrodomain of macroH2A1.1 were performed at 30 °C for 30 mins. Reactions containing NUDT16 were supplemented with 15 mm MgCl_2_ 55.

Enzymatic reaction containing CIP were performed by adding 10U of CIP (NEB) and incubated at 37 °C for 30 mins.

## Author contributions

The experiments were conceived and planned and the manuscript was written by DM and IA; experiments were performed by DM. The final version of this manuscript has been read and approved by all authors.

## Conflicts of interest

The authors declare no competing interests
